# The association of cultural orientation with adherence to social distancing behaviors during the early COVID-19 pandemic in the United States: A cross-sectional survey

**DOI:** 10.1371/journal.pgph.0000866

**Published:** 2022-08-11

**Authors:** Rachel Dinero, Allison Pieczonka, Brittany L. Kmush

**Affiliations:** 1 Department of Psychology, Cazenovia College, Cazenovia, New York, United States of America; 2 Mental Health Counseling Program, St. John Fisher College, Rochester, New York, United States of America; 3 Department of Public Health, Syracuse University, Syracuse, New York, United States of America; National Institute of Neurosciences & Hospital, BANGLADESH

## Abstract

Non-pharmaceutical interventions are one of the major tools to prevent the spread of SARS-CoV-2. Information about these behaviors is disseminated by messaging campaigns. However, people differ in their responses to persuasive messages. Here, we examine whether cultural orientation is associated with adherence to recommended COVID-19 prevention guidelines. Participants (n = 443, 201 from the United States via Amazon Mechanical Turk and 242 from Central New York via a convenience, snowball sample) completed an online survey during April and May 2020. Cultural orientation was measured via the Horizontal and Vertical Individualism and Collectivism Scale. Adherence to limiting social contact was self-reported. Multi-level Poisson regression assessed the association between cultural orientation and social contact behaviors. Those high in horizontal individualist characteristics had a positive association with increased social contact behaviors (RR: 2.20, 95%CI: 1.97–2.47, p<0.001). Those high in vertical collectivist characteristics had a negative association with those behaviors (RR: 0.59, 95%CI: 0.52–0.67, p<0.000). We found an association with cultural orientation and adherence to social contact behaviors during the beginning of the COVID-19 pandemic. In the United States, effective public health messages to promote adherence to preventative behaviors should be tailored to horizontal individualists, those least likely to engage in recommended behaviors.

## Introduction

Non-pharmaceutical interventions are one of the major tools that public health and medical professionals recommend to prevent the spread of SARS-CoV-2. These interventions are largely behavioral and include staying at home, wearing a mask, social distancing, and hand washing. In the absence of laws or mandates, most of these behaviors are voluntary guidelines, with information about the need and efficacy of each behavior disseminated by public health messaging campaigns. However, people differ in their susceptibility and responses to persuasive messages [1–6]. Applying inappropriate strategies can result not only in low adherence rates, but also behavior that is in opposition to that suggested by the message [1–6]. In the United States, preliminary evidence has found that young people, male, political conservatives, and those with less education are less likely to practice COVID-19 prevention behaviors, although more research is needed [7]. People who did not want to participate in these behaviors reported feeling less personally responsible for spread prevention [7]. The goal of the present research is to better understand the underlying factors that shape nonadherence to public health guidelines in the United States. This will allow policy makers to design recommendations surrounding messaging strategies that will be more effective with Americans most likely to be nonadherent.

When looking at global differences in early containment of SARS-CoV-2, European countries (i.e., Italy, Germany, France, Spain) were less effective at containing the initial spread of COVID-19 than Asian countries (i.e., China, South Korea, Japan) [8]. One possible framework for understanding this difference is cultural orientation, specifically those of individualism and collectivism. Hofstede’s cultural model of individualism-collectivism is the most widely referenced model of cultural orientation [9–12]. In individualist cultures, such as the U.S., Canada, and Denmark, individuals are defined by their personal traits and characteristics [13]. Separateness and individuality are valued [13]. There is a drive for uniqueness, differentiation, and separation from others [13]. Individualists are more likely to focus on individual needs over group goals [13]. In collectivist cultures, such as Japan, China, Korea, individuals are defined by their relationships and their place within the group [13]. There is a drive for connectedness, closeness, and acceptance [13]. Value is placed on similarity and fitting in [13]. Collectivism positively predicts mask usage at the group level, with mask usage being higher in countries and U.S. counties that are higher in collectivism [14]. The goal of the present research is to address this association at the individual level within the U.S.

The horizontal/vertical distinction provides additional specificity for defining cultural orientation [15, 16]. Horizontal cultures emphasize equality while vertical cultures emphasize hierarchy [13]. Horizontal individualism (HI) strives for distinctiveness and individuality. Value is placed on independence and self-reliance. While others are seen as equals, the sense of self is distinct and separate [13]. Horizontal collectivism (HC) values interdependence and equality. Others are seen as equals and cooperation and group cohesion are valued [13]. Vertical individualism (VI) strives for distinctiveness and status, value is placed on competition and winning [13]. Vertical collectivism (VC) values interdependence with an acceptance of hierarchy. There is an emphasis on the importance of submission to ingroup authority and sacrifice [13]. Cultural orientation is commonly used to explain differences between countries but can also be measured between individuals using self-report measures [17].

Cultural orientation influences the impact of persuasive strategies, as different persuasive strategies appeal to different types of cultural orientations [18–20]. Individualists are less likely than collectivists to be influenced by persuasive messaging overall, yet more likely to be persuaded by scarcity [18, 19]. That is, placing greater value on things that are in short supply or, making more valuable things less available [18]. Individualists are also less than collectivists likely to be persuaded by authority (messages delivered by experts or people with knowledge or power), reciprocity (messages delivered by those to whom the person is indebted to or owes), liking (messages delivered by likeable people), or consensus (persuasion based on doing what others are doing) [16]. For example magazine advertisements in individualist cultures are more likely to appeal to personal benefits, personal success, pleasure, and independence [16–19]. In collectivist cultures, magazine advertisements are more likely to appeal to harmony, ingroup benefits, and family [16]. Furthermore, magazine advertisements in vertical individualist cultures are more likely to emphasize status, while horizontal individualist cultures are more likely to emphasize uniqueness [19].

Here, we present a cross-sectional study examining the association between adherence to social distancing behaviors aimed at lowering transmission of COVID-19 and cultural orientation on an individual level from April to May of 2020. During this time in the United States, stay at home and social distancing messaging was widespread but mask wearing was not yet widely promoted [21]. The goal of the present research is to identify those who are less likely to engage in protective practices during public health crises in order to develop targeted messaging.

## Methods

### Study design, setting, and participants

Participants were recruited to complete an online survey hosted anonymously through SurveyMethods.com. There were 201 participants recruited through Amazon’s Mechanical Turk, an established method of self-reported data collection [22, 23]. These participants completed the survey on April 29, 2020. An additional 242 participants were recruited through convenience sampling through social media posts and the email list-serve of students and employees at a small rural college in Central New York. The convenience sample was collected from April 29 to May 11, 2020. Participants, all adults, provided written informed consent through the online survey. All study procedures were approved by the Cazenovia College Institutional Review Board.

### Variables

Participants completed an online survey assessing adherence to COVID prevention behaviors, attitudes toward COVID prevention behaviors, psychological characteristics, and cultural orientation. To assess adherence to social distancing behaviors, participants were asked to rate the frequency with which they had done each of the following behaviors in the past two weeks: came within 6 feet of anyone who does not live with you, were part of a small gathering of people who do not live with you, were part of a large gathering of people who do not live with you, used public transportation, and used a taxi or rideshare service. The behaviors were selected based on the CDC guidelines for recommended prevention behaviors at the time of the survey. Participants were instructed to rate the frequency with which they had done each behavior across the past two weeks separately for work, non-work essentials, or leisure for a total of 15 risk behavior items. The frequency of each behavior was rated on a 5-point Likert scale (never, a few of the days, half of the days, most of the days, every day). Some participants responded that they did not leave the house for the specific type of activity (work, essential, or leisure) and were eliminated from any analysis that included those behaviors. General attitude towards adherence to COVID-19 prevention behaviors was measured using a 9-item scale created for this study. Participants used a 5-point Likert scale to rate the importance of 9 behaviors recommended by the CDC to slow or reduce the spread of COVID-19 (e.g., washing hands regularly, avoiding crowded places or large gatherings). Cultural orientation was measured using the validated Horizontal and Vertical Individualism and Collectivism Scale, which assesses Horizontal Individualism (HI), Vertical Individualism (VI), Horizontal Collectivism (HC) and Vertical Collectivism (VC) [24]. The number of new COVID-19 infections the week of April 29, 2020 was retrieved from COVID-19 Data Repository by the Center for Systems Science and Engineering (CSSE) at Johns Hopkins University [25]. Participants with missing data were eliminated from the analysis where that data was used. The populations and population density for the self-reported county of residence of each participant was retrieved from the 2010 US Census [26].

### Statistical methods

The five items for work-related risk were summed to form a work-related risk variable, the five items for essential-related risk were summed, as were the five leisure-related risk variables (possible range of 0–20). All 15 items were summed to form a cumulative risk variable (possible range 0–60). Cultural orientation subscales (i.e., horizontal individualism, vertical individualism, horizontal collectivism, and vertical collectivism) were calculated by averaging the four items pertaining to each subscale. Demographic characteristics comparing the mTurk and convenience samples were completed using Pearson’s χ^2^ test for categorical variables and Student’s T-test for continuous variables. Cultural orientation scores and self-reported social contact behavior were compared between the mTurk and convenience cohorts using Wilcoxon Rank Sum test. The association between social contact and cultural orientation was assessed using multi-level mixed effects Poisson regression to model the counts of the risk-taking behavior, both unadjusted and adjusted for confounders. The cohort (mTurk and convenience sample) was included as the level variable in both adjusted and unadjusted analysis. The adjusted models include age (continuous in years), gender (female, male, non-binary, and no response), ethnicity (Black, Asian, white, Hispanic/LatinX, Persian, multi-ethnic), household income (less than $20,000, $20,000 to $49,000, $50,000 to $99,000, $100,000 to $150,000, over $50,000, and no response), current infection rate in county of residence (new infections per 100,000 population), population density of the county (residents per square mile), and general adherence attitudes (average of 9-items assessing general attitude toward adherence to COVID-19 prevention behaviors, α = 0.94 for mTurk, 0.90 for Convenience, 0.92 combined). Each cohort was also analyzed separately in a sensitivity analysis. Two-sided p-values less than 0.05 were considered statistically significant. Analysis was completed using Stata Version 16 [27]. All data used for this analysis is available is S1 Data.

## Results

There were 443 participants included in this study, 201 completed the survey via Amazon’s mTurk and 242 were part of the convenience sample. The two cohorts were generally different on the demographic characteristic examined (p<0.05) (Table 1). The average age of the participants was 38.0 years (SD: 11.6), ranging from 18 to 75 years, with no difference between the two cohorts (p = 0.22). More males (63.5%) were included in the mTurk cohort whereas more females (87.2%) were included in the convenience cohort (p<0.000) (Table 1). The majority of the participants were white (83.0%) and most had completed a bachelor’s degree or higher (65.4%). Just under half (44.2%) did not see a change in income due to COVID and about a third (33.4%) were working remotely or from home due to COVID at the time of the survey. The mTurk participants were from 40 US states and territories, with less than 10% from any one state or territory except for California (13%). The convenience cohort had participants from 25 US states and territories with 78.1% from New York state, and no more than 3% from any of the other states. Incidence rates of COVID-19 infections in the county were generally low during the time of the study, ranging from 0.0002 to 9.2 per 100,000 population. Ninety eight percent of participants were from counties with less than 1.0 infection per 100,000 people.

**Table 1:** Demographic characteristics of survey participants, United States, April-May 2020.

Demographic Characteristic	MTurk (n = 201)	Convenience (n = 242)	Combined (n = 443)	p-value*
	n (%)	n (%)	n (%)	
Gender				<0.000
Females	70 (34.8%)	211 (87.2%)	281 (63.4%)	
Males	127 (63.2%)	29 (12.0%)	156 (35.2%)	
Non-Binary	1 (0.5%)	2 (0.8%)	3 (0.7%)	
No Response	3 (1.5%)	0 (0.0%)	3 (0.7%)	
Ethnicity/Race				<0.000
Black/African American	11 (5.5%)	1 (0.4%)	12 (2.7%)	
Asian	16 (8.0%)	0 (0.0%)	16 (3.6%)	
Caucasian/White	148 (73.6%)	219 (90.5%)	367 (83.0%)	
Hispanic/Latinx	10 (5.0%)	6 (2.5%)	16 (3.6%)	
Persian	0 (0.0%)	1 (0.4%)	1 (0.2%)	
Multi-Ethnic	4 (2.0%)	9 (3.7%)	13 (2.9%)	
No Response	12 (6.0%)	6 (2.5%)	18 (4.0%)	
Education				<0.000
Some high school	3 (1.5%)	0 (0.0%)	3 (0.7%)	
High school diploma	17 (8.5%)	2 (0.8%)	19 (4.3%)	
Some college	30 (15.0)	56 (23.1%)	86 (19.4%)	
Associates degree	22 (11.0%)	23 (9.5%)	45 (10.2%)	
Bachelor’s degree	101 (50.3%)	58 (24.0%)	159 (35.9%)	
Some graduate work	5 (2.5%)	16 (6.6%)	21 (4.7%)	
Grad/professional degree	23 (11.4%)	87 (36.0%)	110 (24.8%)	
Household Income				<0.000
Less than $20,000	19 (9.5%)	18 (7.4%)	37 (8.4%)	
$20,000 to $49,000	66 (33.8%)	47 (19.4%)	113 (25.5%)	
$50,000 to $99,000	83 (41.3%)	74 (30.6%)	157 (35.4%)	
$100,000 to $150,000	24 (11.9%)	55 (22.7%)	79 (17.8%)	
Over $150,000	6 (3.0%)	34 (14.0%)	40 (9.0%)	
No Response	3 (1.5%)	14 (5.8%)	17 (3.8%)	
Income Change Due to COVID-19**				
Not earning income before COVID-19	8 (4.0%)	19 (7.8%)	27 (6.1%)	0.09
Earning income before COVID-19	87 (43.3%)	107 (44.2%)	194 (43.8%)	0.84
No change in income as a result of COVID-19	88 (43.8%)	108 (44.6%)	196 (44.2%)	0.86
Decrease in income as a result of COVID-19	55 (27.4%)	61 (25.2%)	116 (26.2%)	0.61
Complete loss in income as a result of COVID-19	17 (8.5%)	25 (10.3%)	42 (9.5%)	0.50
Increase in income as a result of COVID-19	6 (3.0%)	15 (6.2%)	21 (4.7%)	0.11
Worked remotely or from home before COVID-19	38 (18.9%)	17 (7.0%)	55 (12.4%)	<0.000
Currently working remotely or from home because of COVID-19	55 (27.4%)	93 (38.4%)	148 (33.4%)	0.01

*Pearson χ^2^ for differences between MTurk and Convenience Samples

**Categories are not mutually exclusive; participants could select all answers that apply.

Seventeen participants (3.8%), all in the mTurk cohort, did not answer the questions about cultural orientation. The mTurk and convenience cohorts were different on all the cultural orientation scores (p<0.05), except for horizontal collectivism (p = 0.85) (Table 2). Seventeen participants (3.8%), all in the mTurk cohort, did not answer the questions about horizontal individualism. Horizontal individualism was approximately normally distributed with a mean of 2.72 (S1 Fig, Table 2). Vertical individualism, horizontal collectivism, and vertical collectivism were all left skewed with means of 3.94, 3.58, and 3.53, respectively (S1 Fig, Table 2). Ten (2.3%) participants did not leave the house for work related activities, three (0.7%) did not leave for essential activities, and six (1.4%) for leisure activities. The mTurk participants reported generally more social contact than the convenience cohort, although these were not statistically significant except for work contact (a mean of 2.57 in mTurk versus 1.21 in the convenience, p = 0.03) (Table 3). The amount of reported social contact was right skewed for all domains examined (S2 Fig).

**Table 2:** Cultural orientation scale scores for survey participants, United States, April-May 2020.

Cultural Orientation	mTurk		Convenience		Combined		p-value*
	n = 201		n = 225		n = 426		
	Mean (sd)	α	Mean (sd)	α	Mean (sd)	α	
Horizontal Individualism	2.82 (1.06)	0.76	2.63 (0.76)	0.60	2.72 (0.92)	0.69	0.03
Vertical Individualism	3.77 (0.74)	0.83	4.09 (0.55)	0.67	3.94 (0.67)	0.76	<0.000
Horizontal Collectivism	3.54 (0.82)	0.70	3.58 (0.71)	0.57	3.56 (0.76)	0.66	0.84
Vertical Collectivism	3.35 (0.87)	0.75	3.69 (0.62)	0.64	3.53 (0.87)	0.69	0.0001

*Wilcoxon Rank Sum test comparing mTurk and Convenience Samples

**Table 3:** Self-reported social contact behaviors of survey participants, United States, April-May 2020.

Social Contact Category	Mturk		Convenience		Combined		p-value*
	Mean (sd)	α	Mean (sd)	α	Mean (sd)	α	
Work Contact**	2.57 (4.54)	0.93	1.21 (2.57)	0.63	1.82 (3.66)	0.88	0.03
	(n = 195)		(n = 238)		(n = 433)		
Essential Contact	2.83 (4.35)	0.94	1.14 (1.02)	0.54	1.01 (3.13)	0.92	0.40
	(n = 199)		(n = 241)		(n = 440)		
Leisure Contact	2.35 (4.37)	0.83	0.74 (1.07)	0.60	1.41 (3.13)	0.93	0.69
	(n = 197)		(n = 240)		(n = 437)		
Cumulative Contact†	7.59 (12.96)	0.98	3.11 (3.21)	0.64	5.11 (9.25)	0.96	0.13
			(n = 235)		(n = 425)		
	(n = 190)						

*Wilcoxon Rank Sum test comparing the mTurk and convenience samples

**Participants were asked to rate the frequency with which they had done each of the following behaviors in the past two weeks: came within 6 feet of anyone who does not live with you, were part of a small gathering of people who do not live with you, were part of a large gathering of people who do not live with you, used public transportation, and used a taxi or rideshare service on a 5-point Likert scale ((0) never, (1) a few of the days, (2) half of the days, (3) most of the days, (4) every day). The answers on the 5 questions were summed to form the category risk.

†Sum of the 3 risk categories.

In the majority of the adjusted Poisson regression models, cultural orientation was associated with social contact (p<0.05), except for the vertical individualists and work contact (p = 0.08) (Table 4). Those high in horizontal individualist characteristics had the strongest association with increased social contact behaviors, with risk ratios ranging from 1.66 (95% CI: 1.51–1.80, p<0.000) for essential contact to 2.20 (95% CI: 1.97–2.47, p<0.001) for leisure contact (Table 4). Vertical individualists and horizonal collectivists had positive, yet somewhat smaller, risk ratios for social contact behaviors (Table 4). Those high in vertical collectivist characteristics were less likely to engage in social contact behaviors with risk ratios ranging from 0.59 (95% CI: 0.52–0.67, p<0.000) for leisure contact to 0.70 (95% CI: 0.63–0.78, p<0.000) for work contact. The same patterns of associations between cultural orientation and social contact behavior were seen in the mTurk cohort (S1 Table). In the convenience cohort, however, the majority of the results were not statistically significant, generally suggesting no association with cultural orientation characteristics and social contact behaviors, except for the association between vertical individualism and overall contact behaviors (RR: 1.26, 95% CI: 1.09–1.46, p = 0.002) (S2 Table).

**Table 4:** Risk ratios for the association between cultural orientation and social contact behaviors (United States, April-May 2020).

		Unadjusted Poisson Regression*				Adjusted Poisson Regression**			
Type of Social Contact	Cultural Orientation	N	RR	95% CI	p-value	N	RR	95% CI	p-value
Work Contact		418				399			
	Horizontal Individualism		1.81	1.67–1.96	<0.000		1.69	1.53–1.86	<0.000
	Vertical Individualism		0.91	0.82–1.00	0.06		1.12	0.99–1.27	0.08
	Horizontal Collectivism		1.00	0.91–1.10	0.99		1.24	1.12–1.39	<0.000
	Vertical Collectivism		0.61	0.56–0.67	<0.000		0.70	0.63–0.78	<0.000
Essential Contact		423				404			
	Horizontal Individualism		1.73	1.60–1.86	<0.000		1.66	1.51–1.80	<0.000
	Vertical Individualism		0.99	0.90–1.10	0.90		1.19	1.05–1.34	0.005
	Horizontal Collectivism		1.01	0.93–1.11	0.78		1.16	1.04–1.28	0.007
	Vertical Collectivism		0.63	0.58–0.68	<0.000		0.70	0.63–0.78	<0.000
Leisure Contact		420				401			
	Horizontal Individualism		2.12	1.94–2.33	<0.000		2.20	1.97–2.47	<0.000
	Vertical Individualism		1.07	0.96–1.21	0.23		1.42	1.23–1.64	<0.000
	Horizontal Collectivism		1.05	0.95–1.17	0.32		1.29	1.14–1.46	<0.000
	Vertical Collectivism		0.54	0.49–0.60	<0.000		0.59	0.52–0.67	<0.000
Cumulative Contact		410				391			
	Horizontal Individualism		1.82	1.74–1.91	<0.000		1.76	1.66–1.87	<0.000
	Vertical Individualism		0.97	0.91–1.03	0.36		1.18	1.09–1.27	<0.000
	Horizontal Collectivism		1.00	0.94–1.05	0.90		1.22	1.14–1.30	<0.000
	Vertical Collectivism		0.60	0.57–0.63	<0.000		0.67	0.63–0.72	<0.000

RR-Risk Ratio; CI-Confidence Interval

*Multilevel, mixed effects Poisson Regression with cohort as a level.

**Multilevel, mixed effects Poisson Regression with cohort as a level, adjusted for age, gender, ethnicity, income, current infection rate in county, population density of county, attitudes towards adherence to guidelines.

## Discussion

This study took place during the very early days of the pandemic, were the most emphasis on preventing COVID-19 was on limiting social contact. Many states had implemented stay-at-home orders for all non-essential workers, but mask mandates had not yet gone into effect [21]. We found a strong association with cultural orientation and social contact behaviors during the beginning of the COVID-19 pandemic. Notably, those high on horizontal individualist traits were more likely to engage in social behaviors considered to put one at risk of contracting COVID-19 while vertical collectivists were the least likely (Table 4). We did not find strong evidence for association between cultural identities and prevention behaviors in the convenience cohort. This is likely because the majority of participants were from New York state, which had very strict lock down measures in place during the time of the survey. This likely made behavior more uniform across the sample, regardless of cultural orientation.

Horizontal individualism, particularly as it was assessed in this survey, emphasizes the cultural value of independence. The horizontal individualism subscale includes items like “I often do my own thing”, and “my personal identity, independent of others is very important to me.” It is not surprising that this is associated with risky social behaviors given the current anti-masking movement in the United States, which places greater importance on civil liberties than public health [28]. While the United States is generally classified as a vertical individualist society, due to the emphasis on competition and independence, it is the drive for independence that predicts risk. Neither the competitive nature of vertical individualism (e.g., “winning is everything”), nor the value on equality and community of horizontal collectivism (e.g., “I feel good when I cooperate with others) are as strongly associated with risky social behaviors as horizontal individualism. The vertical collectivist values of community and submission to authority (e.g., “it is important to me that I respect the decisions made by my group”, unsurprisingly, is the best predictor of risk avoidance.

Only people who left their house during the two weeks prior to the survey reported the frequency of their compliance behaviors. Those who did not leave their house were eliminated from the analysis. The extent of the risky behaviors was modeling using a Poisson distribution, even though the behaviors were not reported as counts but estimated using a Likert scale. The influence of socially desirable responding is a concern for any research that relies on self-report. It should be noted that cultural differences in socially desirable responding have been found, and cultural orientation is associated with different types of socially desirable responding [29]. Given the possibility of socially desirable responding, future research should include observed or documented measures of adherence. Additionally, the sample may not have adequately captured the wide range of behaviors and the spectrum of cultural identities found across the United States, with people from New York state overrepresented from the use of a convenience sample. The small sample size and use of convenience sampling make it difficult to conclusively establish the association between cultural orientation and social contact behaviors. Despite these limitations, this study provides evidence of the associations between individual levels of cultural orientation and risk behaviors. This has significant implications for the types of public health messaging that may be effective in the United States.

The current climate of animosity and hostility towards public health officials has undermined the United States ability to be prepared for the next public health crisis [30]. Public health officials need tools to effectively communicate the reasoning behind laws and recommendations to those most likely to be nonadherent. Since horizontal individualists are the most likely to engage in risk behaviors, effective messages should avoid authority (e.g. avoid “wear a mask because experts or government officials tell you to” or any direct instruction), reciprocity (e.g. avoid “you owe it to your family/neighbors/etc. to protect them”), liking (e.g., no need to use a known or liked figure in the message), consensus (e.g., message should emphasize being an individual not going along with others), ingroup benefits (e.g., message should not focus on the health benefits to others) and family (e.g., message should not include benefits to loved ones), harmony (e.g., message should not include themes of getting along or coming together as a country), fear appeal (e.g., avoid “not wearing a mask will kill you”), and data-driven messaging (e.g., messaging should not include prevalence or risk statistics). The message should include scarcity (e.g., limited edition masks), personal benefits (e.g., promote masks that look and feel good and make a personal statement), personal success (e.g., frame masks in terms of winning and success), and independence (e.g., masks that make a unique statement and are different from what other people have).

As the United States enters another year of the COVID-19 pandemic and policy makers are struggling with opposition to mask and vaccine mandates, it is imperative to craft public health messages that will impact those least likely to adhere to preventative behaviors. Our results demonstrate that horizontal individualists are least likely to adhere to preventative behaviors. Therefore, public health messages should include content that acknowledges the need for independence and does not focus on the group benefits. While this is counter to most current messaging strategies, it could explain the violent reaction to public health officials and their recommendations to prevent COVID-19.

The current reluctance toward vaccination and mask wearing in the United States has striking parallels with the reluctance for many Americans to wear seatbelts. Seatbelts, a proven life saver, have been required to be installed in all cars sold in the United States since 1966 [31]. Despite these measures, people have repeatedly protested wearing seatbelts, arguing that the decision to wear a seatbelt should be a personal choice [32]. In an effort to increase seatbelt usage, the National Ad Council ran numerous ads employing a variety of strategies for over 25 years, encouraging people to “Buckle Up”, without making much impact [32]. Finally, states began to implement laws requiring seatbelt usage and by 1995, all states except New Hampshire had seatbelt laws [32]. Seatbelt laws have increased seatbelt use from 11% in 1981 to almost 85% in 2010, whereas ads made little impact [33]. Therefore, laws enforcing public health behaviors are needed in order to increase adherence, however, the messaging surrounding those laws needs to be specifically crafted to target those least likely to engage in those behaviors.

## Supporting information

S1 Table Risk ratios for the association between cultural orientation and social contact behaviors in the mturk cohort (United States, April-May 2020). RR-Risk Ratio; CI-Confidence Interval. *Poisson Regression with cohort as a level, adjusted for age, gender, ethnicity, income, current infection rate in county, population density of county, adherence general (attitude).
(DOCX)

S2 Table Risk ratios for the association between cultural orientation and social contact behaviors in convenience cohort (United States, April-May 2020). RR-Risk Ratio; CI-Confidence Interval. *Poisson Regression with cohort as a level, adjusted for age, gender, ethnicity, income, current infection rate in county, population density of county, adherence general (attitude).
(DOCX)

S1 Fig Distribution of cultural orientation scores by cohort (United States, April-May 2020). mTurk n = 201; Convenience n = 225.
(DOCX)

S2 Fig Distribution of social distancing behaviors by cohort (United States, April-May 2020). Cumulative: mTurk n = 190, Convenience n = 235; Work: mTurk n = 195, Convenience n = 238; Essential: n = 199, Convenience n = 241; Leisure: mTurk n = 197, Convenience n = 240.
(DOCX)

S1 Data Data and data codebook used for this analysis. (XLSX)
