# Supplementary material for: The association of cultural orientation with adherence to social distancing behaviors during the early COVID-19 pandemic in the United States: A cross-sectional survey
Source: PLOS Glob Public Health. 2022 Aug 11;2(8):e0000866. doi: 10.1371/journal.pgph.0000866 (PMC10021574; doi:10.1371/journal.pgph.0000866)
Supplement: S1 Table — RR-Risk Ratio; CI-Confidence Interval. *Poisson Regression with cohort as a level, adjusted for age, gender, ethnicity, income, current infection rate in county, population density of county, adherence general (attitude). (DOCX) [file pgph.0000866.s001.docx]

**S1 Table.** Risk Ratios for the association between cultural orientation and social contact behaviors in the mTurk Cohort (United States, April-May 2020).

|  |  | Unadjusted Poisson Regression | | | | Adjusted Poisson Regression* | | | |
| --- | --- | --- | --- | --- | --- | --- | --- | --- | --- |
| **Type of Social Contact** | **Cultural Orientation** | **N** | **RR** | **95% CI** | **p-value** | **N** | **RR** | **95% CI** | **p-value** |
| Work Contact |  | 195 |  |  |  | 183 |  |  |  |
|  | Horizontal Individualism |  | 2.16 | 1.96-2.37 | <0.000 |  | 2.33 | 2.04-2.67 | <0.000 |
|  | Vertical Individualism |  | 0.88 | 0.79-0.99 | 0.04 |  | 1.01 | 0.87-1.18 | 0.88 |
|  | Horizontal Collectivism |  | 1.06 | 0.96-1.19 | 0.25 |  | 1.31 | 1.13-1.51 | <0.000 |
|  | Vertical Collectivism |  | 0.56 | 0.50-0.60 | <0.000 |  | 0.63 | 0.55-0.71 | <0.000 |
| Essential Contact |  | 199 |  |  |  | 187 |  |  |  |
|  | Horizontal Individualism |  | 1.94 | 1.78-2.12 | <0.000 |  | 2.00 | 1.79-2.25 | <0.000 |
|  | Vertical Individualism |  | 0.97 | 0.87-1.09 | 0.61 |  | 1.17 | 1.02-1.36 | 0.03 |
|  | Horizontal Collectivism |  | 1.04 | 0.94-1.15 | 0.48 |  | 1.20 | 1.06-1.37 | 0.006 |
|  | Vertical Collectivism |  | 0.56 | 0.51-0.62 | <0.000 |  | 0.62 | 0.54-0.70 | <0.000 |
| Leisure Contact |  | 197 |  |  |  | 185 |  |  |  |
|  | Horizontal Individualism |  | 2.37 | 2.13-2.63 | <0.000 |  | 3.10 | 2.64-3.63 | <0.000 |
|  | Vertical Individualism |  | 1.03 | 0.91-1.17 | 0.62 |  | 1.47 | 1.23-1.74 | <0.000 |
|  | Horizontal Collectivism |  | 1.12 | 1.00-1.26 | 0.06 |  | 1.47 | 1.25-1.71 | <0.000 |
|  | Vertical Collectivism |  | 0.50 | 0.45-0.55 | <0.000 |  | 0.54 | 0.46-0.62 | <0.000 |
| Cumulative Contact |  | 190 |  |  |  | 178 |  |  |  |
|  | Horizontal Individualism |  | 2.10 | 1.99-2.22 | <0.000 |  | 2.32 | 2.14-2.51 | <0.000 |
|  | Vertical Individualism |  | 0.94 | 0.88-1.01 | 0.08 |  | 1.12 | 1.03-1.23 | 0.01 |
|  | Horizontal Collectivism |  | 1.04 | 0.98-1.11 | 0.23 |  | 1.32 | 1.21-1.43 | <0.000 |
|  | Vertical Collectivism |  | 0.54 | 0.51-0.57 | <0.000 |  | 0.60 | 0.56-0.65 | <0.000 |

RR-Risk Ratio; CI-Confidence Interval

*Poisson Regression with cohort as a level, adjusted for age, gender, ethnicity, income, current infection rate in county, population density of county, adherence general (attitude).
