# Supplementary material for: The association of cultural orientation with adherence to social distancing behaviors during the early COVID-19 pandemic in the United States: A cross-sectional survey
Source: PLOS Glob Public Health. 2022 Aug 11;2(8):e0000866. doi: 10.1371/journal.pgph.0000866 (PMC10021574; doi:10.1371/journal.pgph.0000866)
Supplement: S2 Table — RR-Risk Ratio; CI-Confidence Interval. *Poisson Regression with cohort as a level, adjusted for age, gender, ethnicity, income, current infection rate in county, population density of county, adherence general (attitude). (DOCX) [file pgph.0000866.s002.docx]

**S2 Table.** Risk Ratios for the association between cultural orientation and social contact behaviors in Convenience Cohort (United States, April-May 2020).

|  |  | Unadjusted Poisson Regression | | | | Adjusted Poisson Regression* | | | |
| --- | --- | --- | --- | --- | --- | --- | --- | --- | --- |
| **Type of Social Contact** | **Cultural Orientation** | **N** | **RR** | **95% CI** | **p-value** | **N** | **RR** | **95% CI** | **p-value** |
| Work Contact |  | 223 |  |  |  | 216 |  |  |  |
|  | Horizontal Individualism |  | 1.04 | 0.89-1.23 | 0.57 |  | 0.92 | 0.78-1.10 | 0.38 |
|  | Vertical Individualism |  | 0.99 | 0.80-1.23 | 0.93 |  | 1.25 | 1.00-1.57 | 0.05 |
|  | Horizontal Collectivism |  | 0.86 | 0.72-1.01 | 0.07 |  | 1.19 | 0.99-1.43 | 0.06 |
|  | Vertical Collectivism |  | 0.98 | 0.81-1.20 | 0.88 |  | 1.04 | 0.84-1.31 | 0.68 |
| Essential Contact |  | 224 |  |  |  | 217 |  |  |  |
|  | Horizontal Individualism |  | 1.12 | 0.95-1.32 | 0.17 |  | 1.07 | 0.89-1.28 | 0.47 |
|  | Vertical Individualism |  | 1.10 | 0.88-1.38 | 0.42 |  | 1.18 | 0.93-1.51 | 0.18 |
|  | Horizontal Collectivism |  | 0.94 | 0.80-1.12 | 0.52 |  | 1.10 | 0.91-1.35 | 0.33 |
|  | Vertical Collectivism |  | 1.03 | 0.84-1.25 | 0.79 |  | 1.12 | 0.89-1.41 | 0.35 |
| Leisure Contact |  | 223 |  |  |  | 216 |  |  |  |
|  | Horizontal Individualism |  | 1.39 | 1.14-1.70 | 0.001 |  | 1.24 | 1.00-1.54 | 0.05 |
|  | Vertical Individualism |  | 1.31 | 0.98-1.75 | 0.07 |  | 1.35 | 0.99-1.83 | 0.06 |
|  | Horizontal Collectivism |  | 0.86 | 0.70-1.06 | 0.15 |  | 1.00 | 0.78-1.27 | 0.97 |
|  | Vertical Collectivism |  | 0.85 | 0.67-1.08 | 0.18 |  | 0.86 | 0.66-1.13 | 0.28 |
| Cumulative Contact |  | 220 |  |  |  | 213 |  |  |  |
|  | Horizontal Individualism |  | 1.14 | 1.04-1.26 | 0.008 |  | 1.04 | 0.94-1.16 | 0.45 |
|  | Vertical Individualism |  | 1.11 | 0.96-1.27 | 0.15 |  | 1.26 | 1.09-1.46 | 0.002 |
|  | Horizontal Collectivism |  | 0.89 | 0.80-0.99 | 0.03 |  | 1.11 | 0.99-1.25 | 0.08 |
|  | Vertical Collectivism |  | 0.97 | 0.86-1.09 | 0.61 |  | 1.02 | 0.89-1.17 | 0.77 |

RR-Risk Ratio; CI-Confidence Interval

*Poisson Regression with cohort as a level, adjusted for age, gender, ethnicity, income, current infection rate in county, population density of county, adherence general (attitude).
