# Supplementary material for: The association of cultural orientation with adherence to social distancing behaviors during the early COVID-19 pandemic in the United States: A cross-sectional survey
Source: PLOS Glob Public Health. 2022 Aug 11;2(8):e0000866. doi: 10.1371/journal.pgph.0000866 (PMC10021574; doi:10.1371/journal.pgph.0000866)
Supplement: S1 Fig — mTurk n = 201; Convenience n = 225. (DOCX) [file pgph.0000866.s003.docx]

**S1 Fig:** Distribution of Cultural Orientation Scores by Cohort (United States, April-May 2020).


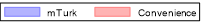


mTurk n=201; Convenience n=225
