# Supplementary material for: The association of cultural orientation with adherence to social distancing behaviors during the early COVID-19 pandemic in the United States: A cross-sectional survey
Source: PLOS Glob Public Health. 2022 Aug 11;2(8):e0000866. doi: 10.1371/journal.pgph.0000866 (PMC10021574; doi:10.1371/journal.pgph.0000866)
Supplement: S2 Fig — Cumulative: mTurk n = 190, Convenience n = 235; Work: mTurk n = 195, Convenience n = 238; Essential: n = 199, Convenience n = 241; Leisure: mTurk n = 197, Convenience n = 240. (DOCX) [file pgph.0000866.s004.docx]

**S2 Fig:** Distribution of Social Distancing Behaviors by Cohort (United States, April-May 2020).

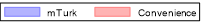


Cumulative: mTurk n=190, Convenience n=235; Work: mTurk n=195, Convenience n=238; Essential: n=199, Convenience n=241; Leisure: mTurk n=197, Convenience n=240.
